# Molecular Detection of *Chlamydia trachomatis* and Other Sexually Transmitted Bacteria in Semen of Male Partners of Infertile Couples in Tunisia: The Effect on Semen Parameters and Spermatozoa Apoptosis Markers

**DOI:** 10.1371/journal.pone.0098903

**Published:** 2014-07-14

**Authors:** Hanen Sellami, Abir Znazen, Afifa Sellami, Hela Mnif, Nour Louati, Soumaya Ben Zarrouk, Leila Keskes, Tarek Rebai, Radhouane Gdoura, Adnene Hammami

**Affiliations:** 1 Department of Microbiology and research laboratory “Microorganismes et Pathologies Humaines”, Habib Bourguiba University Hospital of Sfax, Sfax, Tunisia; 2 Histology Embryology Research Unit, Faculty of Medicine of Sfax, University of Sfax, Sfax, Tunisia; 3 Sfax Regional Center of Blood Transfusion, Sfax, Tunisia; 4 Unit Research of Toxicology-Microbiology Environmental and Health, Sciences Faculty of Sfax, University of Sfax, Sfax, Tunisia; University of Missouri-Kansas City, United States of America

## Abstract

This study was undertaken to determine the prevalence of *Chlamydia trachomatis*, *Mycoplasmas*, and *Ureaplasmas* in semen samples of the male partners of infertile couples and to investigate whether *Chlamydia trachomatis* could initiate apoptosis in human spermatozoa. A total of 85 males partners of infertile couples undergoing routine semen analysis according to World Health Organization guidelines were included. Specimens were examined for the presence of *Chlamydia trachomatis*, *Neisseria gonorrhoeae*, *Mycoplasma hominis*, *Mycoplasma genitalium*, *Ureaplasma urealyticum* and *Ureaplasma parvum* by Real time PCR (qPCR). Semen specimens were analysed for the appearance of apoptotic markers (sperm DNA fragmentation, activated caspase 3 levels, mitochondrial membrane potential (ΔΨm)) using flow cytometry. *C. trachomatis*, *N. gonorrhoeae*, *U. urealyticum*, *M genitalium* were detected in semen samples of 13 (15.2%), 5 (5.8%), 5 (5.8%) and 3 (3.5%) male partners of infertile couples, respectively. *M. hominis* and *U. parvum* were detected in semen sample of only one patient (1.1%). The semen of infertile men positive for *C. trachomatis* showed lower mean of semen count and lower rapid progressive motility (category [a]) of spermatozoa compared to uninfected men with statistically significances (*p* = 0.02 and *p* = 0.04, respectively). Flow cytometry analyses demonstrated a significant increase of the mean rate of semen with low ΔΨm and caspase 3 activation of infertile men positive for *C. trachomatis* compared to uninfected men (*p* = 0.006 and *p* = 0.001, respectively). DNA fragmentation was also increased in sperm of infertile men positive for *C. trachomatis* compared to uninfected men but without statistical significances (*p* = 0.62). *Chlamydial* infection was associated to loss of ΔΨm and caspase 3activation. Thus, *C*. *trachomatis* infection could be incriminated in apoptosis induction of spermatozoa. These effects may explain the negative direct impact of *C. trachomatis* infection on sperm fertilizing ability.

## Introduction

Sexually transmitted infections are of major concern to researchers and clinicians in the field of reproductive medicine. It is estimated that 15% of male infertility is related to genital tract infection [1]. Men can harbor subclinical infections in the genital tract over extended periods of time and several sexually transmitted infection pathogens, such as *C. trachomatis* have been detected in semen from asymptomatic men [2]. According to a World Health Organization (WHO) [3] report, *C. trachomatis* is responsible for the most common sexually transmitted bacterial infection worldwide, affecting more than 90 million people and has been known for some time to have a significant effect on human reproduction [4]. The role of *C. trachomatis* infections in male infertility is controversial [5]–[6]. A number of studies have specifically looked at the relationship between *Chlamydial* infection and semen quality. While some authors have shown that *C. trachomatis* infection is associated with poor semen quality [7]–[8], others have claimed that it does not [9]–[10]. Some reports indicated that *C. trachomatis* infection is associated with a decrease in sperm concentration and motility and also with altered semen pH and reduced volume of the ejaculate [11], [12], [13]–[14]. Conversely, other studies have revealed no association between *C. trachomatis* infection of the male genital tract and altered sperm quality [9], [15], [16], [17], [18], [19], [20]–[21]. In summary, the available evidence is conflicting and still makes it impossible to establish a clear relationship between *C. trachomatis* infection and semen quality.

The apoptotic mode of cell death is an active and defined process which plays an important role in the development of multicellular organisms and in the regulation and maintenance of the cell populations in tissues upon physiological and pathological conditions [22]. Apoptosis markers characterized in somatic cells were noted in human spermatozoa in several studies. These include, principally, plasma membrane externalization of phosphatidylserine (PS) and DNA fragmentation. Such markers are observed with higher frequency in ejaculates of infertile men compared with fertile controls [23]–[24]. In addition, key components of the somatic cell apoptosis pathways, such as presence and activation of caspases, have been described in purified populations of ejaculated sperm from the high and low-motility fractions [24]–[25]. Moreover, mitochondria play a major role in the control of apoptosis [26]. Marchetti *et al* (2002) demonstrated that analysis of ΔΨm is a sensitive test to determine sperm quality when compared with the analysis of the basic sperm parameters, generation of reactive oxygen species, and presence of DNA fragmentation [27]. Several *in vitro* and *in vivo* studies tried to establish a relationship between apoptosis markers in spermatozoa and *Chlamydial* infection. *In vitro,* some authors have demonstrated that *C. trachomatis* is able to interact with sperm cells, affecting their function and inducing apoptosis [28], [29]–[30]. Apoptosis of human sperm can be induced by *in vitro* incubation of human sperm cells with *Chlamydial* LPS, which has a 550 fold greater spermicidal activity than *Escherichia coli* LPS [31]–[32]. In addition, *C. trachomatis* serovar E can attach to human spermatozoa and influence its function leading to premature capacitation [33]. It has been shown that *Chlamydial* LPS interact with CD14 on the sperm surface, thus leading to increased production of reactive oxygen species and resulting in caspase-mediated apoptosis [29]. Despite all this *in vitro* studies, a clear association between *C. trachomatis* and sperm damage has not yet been corroborated by *in vivo* studies. Gallegos *et al* (2008) reported that patients with *C. trachomatis* and *Mycoplasmas* genitourinary infections have increased sperm DNA fragmentation in comparison with fertile controls [34]. Lastly, we showed that inoculation of fertile male Swiss mice in the meatus urethra with *C. trachomatis* could lead to alteration of semen parameters, induction of apoptosis in spermatozoa, and decrease of the reproductive performance of male mice [35]. Taken together, these data support a role of *C. trachomatis* in sperm apoptosis induction. However, most studies indicate that apoptosis-inducing mechanism is unknown.

In the present Study, we aimed to determine the prevalence of *C. trachomatis*, *Mycoplasmas*, and *Ureaplasmas* in semen samples of the male partners of infertile couples and mainly to investigate whether *C. trachomatis* could initiate apoptosis in human spermatozoa.

## Materials and Methods

### Subjects

A total of 85 infertile men attending obstetrics and gynecology clinics in Sfax (South of Tunisia) for diagnostic semen analysis were selected to the study. All men were undergoing semen analysis as part of a work-up for infertility investigations after failing to conceive with their partner after one year of unprotected intercourse. The mean duration of infertility was 4 years (range 1–15). The mean age of patients was 36.7 years (range 23–57). This study was approved by our institutional review board “Habib Bourguiba University hospital ethics committee” with the given number 8–12. All subjects signed a written informed consent. Consent form was also approved by our ethic committee

### Sperm seminological variables

Prior to semen analysis, the men were asked to abstain from sexual intercourse or masturbation for 3–5 days before attending the clinic. All samples for analysis were produced on site and collected into standard containers that had previously been shown not to have any cytotoxic effects on human spermatozoa according to the methods outlined by WHO. Immediately after semen production, samples were placed in an incubator and liquefied at 37°C for up to 30 minutes before analysis. Semen analysis was performed according to the WHO criteria [36] to determine the following variables: sperm concentration, vitality, total progressive motility (category [a+b]), rapid progressive motility (category [a]) Peroxidase staining, a practical and reliable method recommended by WHO [36] for determining leukocytes in the semen, was employed to count and differentiate leukocytes (white blood cells) from immature germ cells. Leukocytospermia was indicated by a concentration of leukocytes ≥10^6^/ml.

### Spermiocultures analysis

Samples were seeded quantitatively using a calibrated loop on agar plates: blood agar, chocolate agar with isovitalex (1%) incubated in 5% CO2 at 37°C for 48 hours. Microorganisms were identified by Gram staining and Bio-Mérieux Api systems (Bio-Mérieux, Marcy l'Etoile, France).

Spermiocultures were considered positive when the number of colonies was ≥10^4^ CFU ml^−1^ in case of Gram positive cocci and ≥10^5^ CFU ml^−1^ in case of Gram negative rods.

### Bacterial quantification in semen specimens by qPCR

For each male patient, 200 µl of semen specimens were used for bacterial quantification by Real time PCR.

#### Extraction of DNA by Cetyltrimethylammonium bromide (CTAB)-phenol-chloroform/isoamyl alcohol method

The precipitates from each 200 µl of semen specimens were harvested by centrifugation at 14,000 g for 20 minutes. The precipitates were treated with 5 µl of proteinase K (20 mg/ml) at 55°C for 2 h in 600 µl of digestion buffer (30 µl of 10% sodium dodecyl sulphate and 570 µl of TE buffer [10 mM Tris-HC1 (pH: 8), 1 mM EDTA]).

After homogenisation, the samples were incubated in a solution of CTAB-NaCl (100 µl of 5 M NaCl and 80 µl of 10% CTAB) for 10 minutes at 65°C, and then mixed with 750 µl of chloroform-isoamyl alcohol (24∶1 [vol/vol]) and centrifuged for 15 minutes at 14,000 g in an Eppendorf centrifuge. The aqueous phase was separated, mixed with 750 µl of phenol chloroform/isoamyl alcohol (25∶24∶1 [vol/vol/vol]) and centrifuged for 15 minutes at 14,000 g in an Eppendorf centrifuge. The obtained aqueous phase was mixed with an equal volume of isopropanol.

The samples were left at −80°C for 1 h and then centrifuged for 15 minutes at 14,000 g. The DNA pellet was washed up once with 70% ethanol, air dried, and dissolved in a final volume of 100 µl of TE buffer.

#### Primers and probes for Qpcr

Initially, the extracted DNA was tested for human β-globin gene to check that there were no PCR inhibitors in the samples. Primers β-GPCO (5′-ACACAACTGTGTTCACTAGC- 3′) and β-GPCPO (5′-GAAACCCAAGAGTCTTCTCT- 3′) were used to amplify a 209-bp fragment of the human β-globin gene [37]. Samples found to be negative by PCR for β-globin were retested after dilution 10-fold in distilled water. Samples shown to be β-globin positive were then examined for bacterial quantification by Real time PCR.

The real-time PCR assay was performed on a CFX96™ real-time PCR cycler (Biorad, USA) in a 20 µl final volume with Ex Taq Premix Tli RNaseH Plus (Takara, Japan). A pair of primers and a labeled probe in the TaqMan format was used to amplify: 149 bp region of Cryptic plasmid for C. trachomatis, 80 bp of MgPa region of Adhesin gene for M. genitalium, 101 bp region of the 16 S rRNA-encoding gene for M. hominis, 101 bp region of Por A pseudogene for N. gonorrhoeae and 146 bp of the Urease gene of U. parvum and U. urealyticum.

Real-time PCR included initial denaturation at 95°C for 2 min, followed by 40 cycles of 95°C for 30 s and annealing temperature according to microorganisms for 30 s (*C. trachomatis* 60°C, *M. genitalium*, *M. hominis* and *N. gonorrhoeae* 55°C, *U. parvum* and *U. urealyticum* 50°C).

In all experiments, each PCR run included a negative extraction control (sterile water) and a negative PCR control, containing 5 µl Diethylpyrocarbonate (DEPC) treated H2O instead of DNA extract, to detect any possible contaminating DNA. Samples and controls were run in duplicate.

#### Positive recombinant plasmid control

To facilitate bacterial quantification, a plasmid containing the target gene for all bacteria was constructed.

DNA was extracted from *C. trachomatis*, *N. gonorrhoeae*, *M. genitalium*, *M. hominis*, *U. parvum* and *U. urealyticum* references strains and the target sequence for all genes selected for Real Time PCR were amplified with the same primers in (Table 1).

**Table 1:** Primers and probes used for detection and quantification of C. trachnomatis, N. gonorrhoeae. U. urealyticum, M. genitalium, U. parvum and M. hominis by qPCR.

Bacteria	Primers and probes	Oligonucleotide sequence (5′→3′)	Target gene	Product size (bp)	Ref
*C. trachnomatis*	Forward	AACCAAGGTCGATGTGATAG	Cryptic plasmid	149	[73]
	Reverse	TCAGATAATTGGCGATTCTT			
	Probe	**ROX**-CGAACTCATCGGCGATAAGG- **BHQ2**			
*N. gonorrhoeae*	Forward	CCGGAACTGGTTTCATCTGATT	PorA	101	[74]
	Reverse	GTTTCAGCGGCAGCATTCA			
	Probe	**FAM**-CGTGAAAGTAGCAGGCGTATAGGCGGACTT-**BHQ1**			
*M. genitalium*	Forward	GAGAAATACCTTGATGGTCAGCAA	MgPa	80	[75]
	Reverse	GTTAATATCATATAAAGCTCTACCGTTGTTATC			
	Probe	**HEX**-ACTTTGCAATCAGAAGGT-**BHQ1**			
*M. hominis*	Forward	TTTGGTCAAGTCCTGCAACGA	16S rRNA-encodinggene	101	[76]
	Reverse	CCCCACCTTCCTCCCAGTTA			
	Probe	**ROX**-TACTAACATTAAGTTGAGGACTCTA-**BHQ1**			
*U. urealyticum*	Forward	CATTGATGTTGCACAAGGAG	Urease (UreD Subunit)	146	[77]
	Reverse	CGTGATTTTAATGTATCGGCTTTC			
	Probe	**FAM** TTGACCACCCTTACGAG **BHQ1**			
*U. parvum*	Forward	CATTGATGTTGCACAAGGAG	Urease (UreD Subunit)	147	[77]
	Reverse	CGTGATTTTAATGTATCGGCTTTC			
	Probe	**Hex** TTGTCCGCCTTTACGAG **BHQ1**			

The final 25 µl reaction mixture contained 1X PCR buffer (Promega, Lyon, France), 0.2 mM each primer, 0.2 mM each dNTP, 2.5 mM MgCl_2_, 1.25 U Go Taq DNA polymerase (Promega), and 5 µL of DNA extract. PCR was performed in Gene-Amp PCR System 9700 (Applied Biosystems, Foster City, California) according to the following procedure: 4 min at 95°C, 35 cycles at 95°C for 30 s, 55°C for 1 min, 72°C for 20 min. PCR products were then purified with QIAquick Gel Extraction Kit (Qiagen) and cloned into a vector using a cloning kit (pGEM-T vector; Promega, Madison, WI, USA), in accordance with the manufacturer's instructions. Isolation of recombinant plasmid DNA was performed using the QIAprepSpin Miniprep kit (Qiagen), and the presence of the correct insert was confirmed by sequencing using the commercial BigDye Terminator v3.1 kit (Applied Biosystems) on a 3730XL sequencer (Applied Biosystems). The obtained sequences were processed by the ABI 3100 Genetic Analyzer and were compared with the sequences available in GenBank by using the BLAST server from the NCBI website (http://www.ncbi.nlm.nih.gov/BLAST). Plasmids were then linearized and quantified with a NanoDrop ND-1000 Spectrophotometer. Copy numbers of the cloned gene was calculated using the following equation reported by [38] to generate standards ranging from 1 to 10^6^ molecules and stored at −20°C.

### Evaluation of Viability of sperm using 7-amino-actinomycin-D Dye

The percentage of dead sperms cells (cells with 7-AAD positive) and viable sperm cells (cells with 7-AAD negative) were assessed using 7-AAD Dye. 7-AAD penetrates only dead cells. From each sperm sample, 1 ml of a sperm solution in PBS containing 2×10^6^cells/ml was stained with 10 µl of 7-amino-actinomycin-D (7-AAD) (Immunotech, a Beckman Coulter Company, Marseille–France). The samples were incubated in the dark at room temperature for 20 minutes before flow cytometric analysis. After the incubation period, 1 ml PBS was added and the sample was analyzed by flow cytometry.

### Evaluation of Mitochondrial Membrane Potential (ΔΨm)

JC-1 possesses the unique ability to differentially label mitochondria with low and high ΔΨm. In mitochondria with high ΔΨm, JC-1 forms multimeric aggregates that emit in the high orange wavelength of 590 nm when excited at 488 nm. In mitochondria with low ΔΨm, JC-1 forms monomers; these monomers emit in the green wavelength (525–530 nm) when excited at 488 nm.

The ΔΨm was analyzed using MitoProbe JC-1 Assay kit (Molecular Probes, Eugene, OR). For staining, 2 µM stock solution of JC-1 in dimethylsulfoxide (DMSO) was prepared. From each sperm sample, 1 ml of a sperm solution in PBS containing 2×10^6^cells/ml was stained with 10 µl of JC-1 stock solution. The samples were incubated at 37°C in the dark for 20 minutes before flow cytometric analysis. In this way, 2 sperm subpopulations were identified:

1. Represented spermatozoa with high ΔΨm (orange fluorescence).
2. Represented spermatozoa with low ΔΨm (green fluorescence).

As suggested by the protocol, in order to confirm the JC-1 sensitivity to changes in membrane potential, carbonylcyanide 3-chlorophenylhydrazone (CCCP  = 50 µM final concentration) was used as membrane potential disruptor (negative control).

### Flow cytometric detection of activated caspase 3

Activated Caspase 3 levels were detected in spermatozoa using fluorescein- labeled inhibitor of caspases (FLICA), which is cell permeable, non cytotoxic, and binds covalently to active Caspase 3. The inhibitor was used with the appropriate controls according to the kit instructions provided by the manufacturer (Carboxyfluorescein FLICA Apoptosis Detection Kit, AbCys, France). Briefly, 3.10^6^ sperm were resuspended in 300 µl PBS. A 150-fold stock solution of the inhibitor was prepared by dissolving the lyophilized caspase-inhibitor in 50 µl dimethyl sulfoxide (DMSO) and was further diluted 1∶5 in PBS to yield a 30-fold working solution (per aliquot: 2 µl of the stock solution plus 8 µl PBS). All test aliquots and controls (with 300 µl PBS) were incubated at 37°C in the dark for 1 h with 10 µl of the working solution. Sperm samples were then washed resuspended in 400 µl of Wash Buffer and kept in ice until flow cytometry analysis.

A negative control (sample with 300 µl PBS) and a positive control (sample treated with 10 µM H_2_O_2_ for 1 hour at 37°C) were used in all experiments.

### TUNEL assay

For the evaluation of DNA fragmentation, a commercial kit (In situ Cell Death Detection Kit, Fluorescein, Takara, Japon) based on an enzymatic reaction of labelling free 3′-OH termini was used. In brief, 3.10^6^ cells were washed with phosphate- buffered saline (1xPBS, pH 7.4) then fixed with 200 µl of 4% paraformaldehyde for 1 h at room temperature in the dark. After wards, sperm cells were washed with 1xPBS and permeabilised using 0.1% Triton X-100 in 0.1% sodium citrate for 15 min on ice. After washing with PBS, sperm DNA was labelled by incubating spermatozoa with 50 µl of the TUNEL reaction mixture (Tdt enzyme and FITC-labelled nucleotides) in a humidified atmosphere for 60 min at 37°C in the dark, with mixing each 15 min. Washed and labelled sperm cells were then resuspended in 1xPBS for flow cytometry analysis. A negative control (sample without the addition of Tdt enzyme) and a positive control (sample treated with DNase I (3 U/ml, Invitrogen) for 10 min at room temperature to generate DNA strand breaks) were also assessed by TUNEL assay.

### Flow Cytometry and data analyses

**Figure 1:**
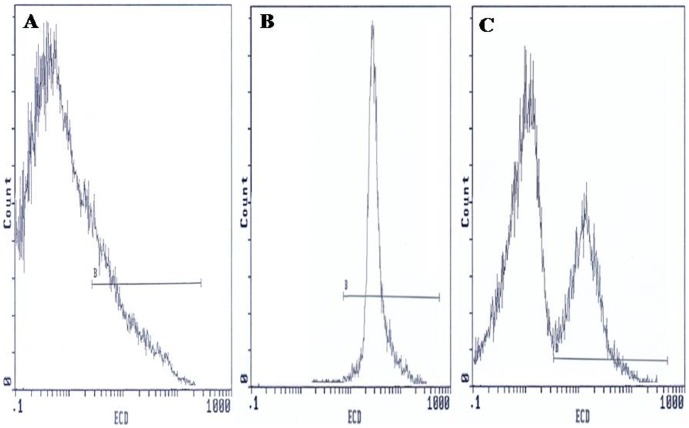
Flow cytometric of sperm viability using 7-amino-actinomycin-D Dye. Histograms show: (**A**) Negative control with 10% 7-AAD positive cells. (**B**) Positive control with 98.5% 7-AAD positive cells. (**C**) Semen sample of one male partner of infertile couples positive for *C. trachomatis* qPCR with 56.5% 7-AAD negative cells and 43.5% 7-AAD positive cells. B: window adjusted to detect the percentage of cells with 7-AAD positive.

**Figure 2:**
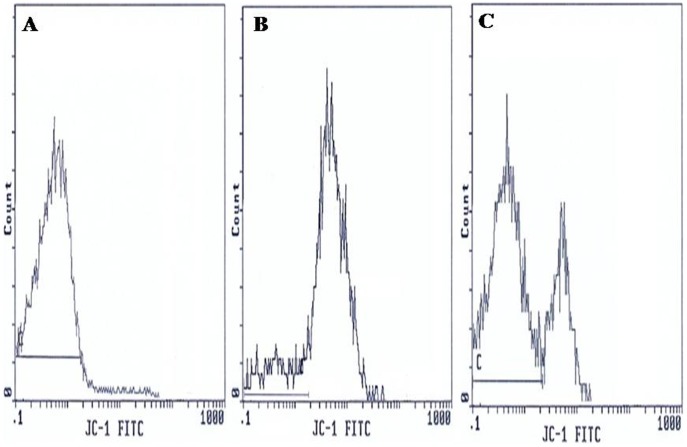
Flow cytometric of changes in the mitochondrial membrane potential (ΔΨm). Histograms show: (**A**) Negative control with 95.8% low ΔΨm cells. (**B**) Positive control with 9.6% low ΔΨm cells. (**C**) Semen sample of one male partner of infertile couples positive for *C. trachomatis* qPCR with 32.5% low ΔΨm cells and 67.5% with high ΔΨm cells. C: window adjusted to detect the percentage of cells with low ΔΨm.

**Figure 3:**
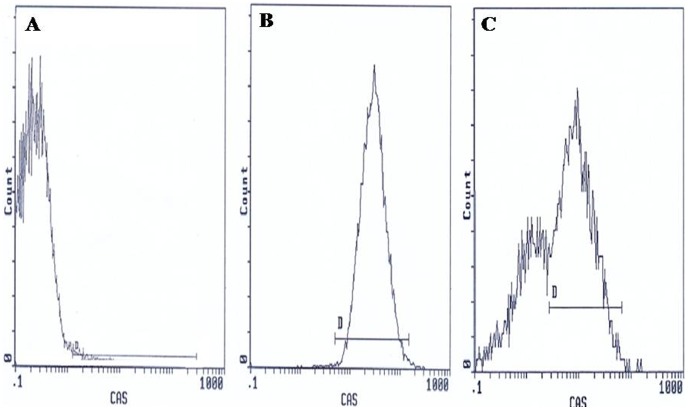
Flow cytometric caspase 3 detection histograms. (**A**) Negative control with 0.85% FITC labelled cells. (**B**) Positive control with 95.8% FITC labelled cells. (**C**) Semen sample of one male partner of infertile couples positive for *C. trachomatis* qPCR with 32.5% FITC labelled cells. D: window adjusted to detect the percentage of cells exhibiting caspase 3 activation.

**Figure 4:**
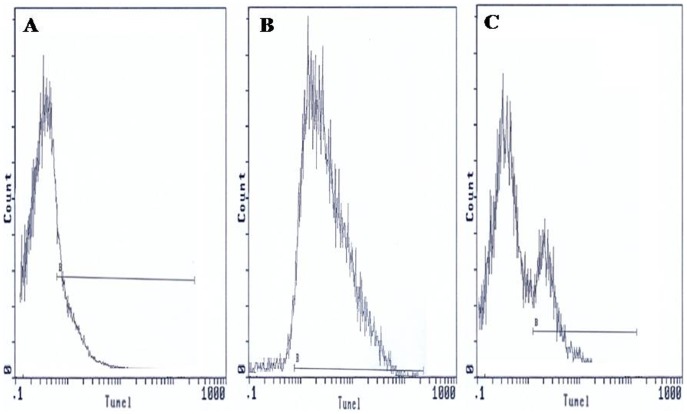
TdT (terminal deoxynucleotidyl transferase)-mediated dUTP nick-end labeling (TUNEL) assay of spermatozoa. Histograms show: (**A**) negative control with 2.35% TUNEL positive cells. (**B**) Positive control (spermatozoa treated with DNaseI) with 90.5% TUNEL positive cells. (**C**) Semen sample of one male partner of infertile couples positive for *C. trachomatis* qPCR with 20.5% TUNEL positive cells. B: window adjusted to detect the percentage of TUNEL positive cells.

Flow cytometric analysis was carried out using an EPICS XL flow cytometer (Beckman Coulter) equipped with a 15mW argon-ion laser for excitation at 488 nm. At least 10,000 events per sample were analysed. Light-scattering and fluorescence data were obtained at a flexed gain setting in logarithmic mode. Debris was excluded by establishing a region around the population of interest on the basis of light scatter characteristics (forward-angle light scatter (FSC) vs. side-angle light scatter (SSC). The percentage of labelled sperm was characterized by identifying a region that included >90% of events in the frequency histogram of the positive controls both in the assessments of Viability, ΔΨm, Caspase 3 activation and DNA fragmentation. Data were expressed as percentage of stained cells from histograms using System II software. Typical examples of histograms obtained by flow cytometry for the detection of fluorescence are shown in Figure 1 (sperm viability), Figure 2 (ΔΨm), Figure 3 (Caspase 3 activation) and Figure 4 (TUNEL assay).

### Statistical analysis

The SPSS 18.0 software (SPSS Inc, Chicago, Ill) was used for statistical analysis. Test χ^2^ was used to compare frequencies. Non-parametric test (Mann-Whitney) from SPSS software was used to compare distribution sperm parameters and flow cytometry data of infected and uninfected men. Correlation between semen parameters means, ΔΨm, DNA fragmentation and caspase 3 activation and *C. trachomatis* infection was assessed using T-test. All tests were considered statistically significant when p<0.05.

## Results

### Spermiocultures analysis

Spermioculture analysis was positive in 6 cases (7%). Group B *Streptococcus* (GBS) was found in 3 samples (3.5%), *Enterococcus spp* in 1 sample (1.1%), *Staphylococcus aureus* in 1 sample (1.1%) and *Corynebacterium spp* in 1 sample (1.1%).

### Frequency of urogenital bacteria in semen samples using qPCR

Among 85 semen samples, 13 (15.2%) were positive for *C. trachomatis* and 5 (5.8%) for *N. gonorrhoeae*. *U. urealyticum, M. genitalium, U. parvum and M. hominis* were detected in 5 patients (5.8%), 3 patients (3.5%), 1 patient (1.1%) and 1 patient (1.1%) respectively. The distribution of detected species in patients is shown in table 2.

**Table 2:** Frequency of urogenital bacteria detected by qPCR and spermiocultures analysis in semen samples of 85 infertile male patients.

Species	Patients N = 85	Frequency (%)
**qPCR**		
*C. trachomatis*	13	15.2
*N. gonorrhoeae*	5	5.8
*M. genitalium*	3	3.5
*M. hominis*	1	1.1
*U. urealyticum*	5	5.8
*U. parvum*	1	1.1
**Spermiocultures**		
Group B *Streptococcus*	3	3.5
*Staphylococcus aureus*	1	1.1
*Enterococcus spp*	1	1.1
*Corynebacterium spp*	1	1.1

### *C. trachomatis* infection and semen quality

The mean values (±SD) for semen parameters of the 85 included patients are shown in Table 3. The sperm vitality and total motility of spermatozoa in the male partners of infertile couples with *C. trachomatis* DNA in semen specimens were lower but not significantly that those of uninfected male partners (71.3% vs 73.3%, *p* = 0.65 and 41.1% vs 43.9 %, *p* = 0.39, respectively) (Table 3). The sperm concentration and rapid progressive motility (category a) of spermatozoa in *C. trachomatis* DNA positive semen were significantly lower than those of uninfected semen (41.4×10^6^/ml vs 84.4×10^6^/ml, *p* = 0.02 and 8.8% vs 12.6%, *p* = 0.04, respectively) (Table 3). The leukocyte count in the male partners of infertile couples with *C. trachomatis* DNA in semen specimens was higher but not significantly than those uninfected semen (0.8×10^6^/ml vs 0.4×10^6^/ml, *p* = 0.36) (Table 3).

**Table 3:** Seminological variables of semen of *C. trachomatis* positive patients compared to uninfected patients.

Variables	Total Semen	Uninfected Semen	*C. trachomatis* positive semen	*p* value#
	n = 85	n = 57	n = 13	
Sperm concentration (x10^6^/ml)	71.1±60.1	84.4±64.6	41.4±42.7	**0.02**
Vitality (%)	72.4±14.7	73.3±14.7	71.3±16.4	0.65
Total progressive motility (category [a+b]) (%)	41.2±12.2	43.9±10.6	41.1±10.4	0.39
Rapid progressive motility (category [a]) (%)	10.8±7.4	12.6±7	8.8±5.4	**0.04**
Leukocyte count (x10^6^/ml)	0.55 ±1.4	0.4±1.5	0.8±1.2	0.36

Note: Values are means (± Standard Error).

# Unless indicated, variables were tested T-Test.

### Sperm viability using 7-amino-actinomycin-D Dye

Figure 1 presents frequency distribution histograms of negative control (Fig. 1A), positive control (Fig. 1B), and one semen of male partners of infertile couples positive for *C. trachomatis* qPCR (Fig. 1C). The percentages of viable sperm cells (cells with 7-AAD negative) were assessed in semen specimens of male partners of infertile couples positive for *C. trachomatis* qPCR and uninfected men. The mean proportion of viable spermatozoa (±SD) in uninfected patients was 63.2±13.9%, while it decreased to 51.3±21.13% in patients positive for *C*. *trachomatis* qPCR with a statistically significant difference (*p* = 0.014) (Table 4).

**Table 4:** 7-AAD, ΔΨm, caspase 3 activation and sperm DNA fragmentation of semen of *C. trachomatis* positive patients compared to uninfected men.

Parameters	Uninfected Semen	*C. trachomatis* positive semen	*p* value#
	n = 57	n = 13	
**Negative 7-AAD (%)**	63.2±13.9	51.3±21.1	**0.014**
**Low ΔΨm (%)**	24.5±9.7	33.7±13.3	**0.006**
**CP 3 activation (%)**	20.8±14	54.5±18.1	**<0.001**
**DNA fragmentation (%)**	25.1±14.3	29.2±17.2	0.62

Values are means (± Standard Error).

# Unless indicated, variables were tested by T-Test.

7-AAD: 7-amino-actinomycin-D.

ΔΨm: Mitochondrial membrane potential.

CP3: Caspase3.

### Mitochondrial Membrane Potential (ΔΨm)

Analysis of the state of mitochondrial respiration in human spermatozoa was assessed using JC-1 to determine the ΔΨm as shown in Figure 2. Flow cytometry results are expressed as percentage of sperm cells with low ΔΨm (green fluorescence). Figure 2 presents frequency distribution histograms of negative control (Fig. 2A), positive control (Fig. 2B), and one semen of male partners of infertile couples positive for *C. trachomatis* qPCR (Fig. 2C). The mean percentage of spermatozoa with low ΔΨm (±SD) was higher in male partners of infertile couples positive for *C. trachomatis* qPCR than those of uninfected patients (33.7±13.3% vs. 24.5±9.7%) and the difference was statistically significant (*p* = 0.006) (Table 4).

### Caspase 3 activation

The results of flow cytometry are expressed as percentage of activated caspase 3 sperm cells. Figure 3 presents frequency distribution histograms of negative control (Fig. 3A), positive control (Fig. 3B), and one semen of male partners of infertile couples positive for *C. trachomatis* qPCR (Fig. 3C). Mean percentage of spermatozoa with activated caspase 3 (±SD) was higher in male partners of infertile couples positive for *C. trachomatis* qPCR than those of uninfected patients (54.5 ±18.1% vs. 20.8±14%) and the difference was statistically significant (*p* = <0.001) (Table 4).

### DNA fragmentation

TUNEL coupled flow cytometry results are expressed as percentage of DNA fragmented sperm cells. Figure 4 presents frequency distribution histograms of negative control (Fig. 4A), positive control (Fig. 4B), and one semen of male partners of infertile couples positive for *C. trachomatis* qPCR (Fig. 4C). Mean percentage of spermatozoa with DNA fragmentation (±SD) was higher in male partners of infertile couples positive for *C. trachomatis* qPCR than those of uninfected patients (29.2±17.2% vs. 25.1±14.3%). But the increase in sperm DNA fragmentation remains statistically not significant (*p* = 0.62) (Table 4).

**Figure 5:**
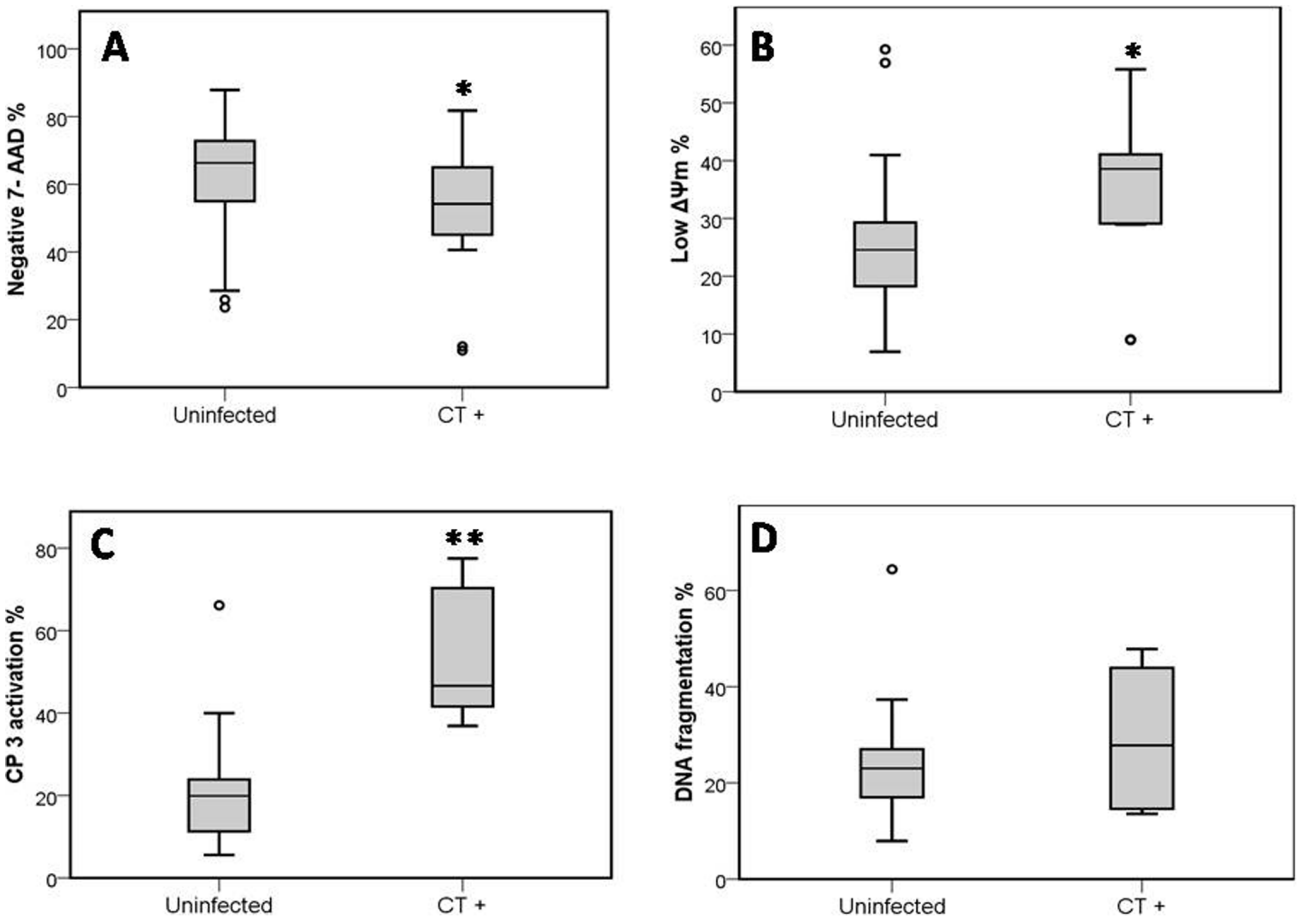
Distributions of percentages of different apoptotic markers among patients positive for *C. trachomatis* qPCR compared to uninfected patients. (**A**) Mean percentage of Sperm Vitality, evaluated with 7-amino-actinomycin-D Dye (7-AAD). (**B**) Mean percentage of Sperm mitochondrial membrane potential (ΔΨm), evaluated with JC-1. (**C**) Mean percentage of Caspase 3 activation, evaluated with fluorescein-labeled inhibitor of caspases (FLICA). (**D**) Mean percentage of Sperm DNA fragmentation, evaluated with (TUNEL). **Uninfected**: Sperm of uninfected patients (negative for all PCRs performed and for spermioculture analysis). **CT^+^**: sperm of patients positive for *C. trachomatis* qPCR. * Indicates significant differences compared with uninfected semen (*P*<0.05). ** Indicates significant differences compared with uninfected semen (*P*<0.001).

Distributions of percentages of different apoptotic markers among patients positive for *C. trachomatis* qPCR compared to uninfected patients are shown in figure 5.

## Discussion

The importance of genital tract microorganisms as an etiologic factor in male infertility is still a controversial topic [39]. The purpose of this study was to determine the prevalence of several common sexually transmitted pathogens among male members of infertile couples. Asymptomatically infected individuals may carry lower amount of organisms [40]. Besides, real time PCR is easier and has higher sensitivity and specificity. Thus, real time PCR may be the technique of choice for bacterial detection and quantification in semen specimens of asymptomatic male partners. Our study demonstrated that *C. trachomatis* seems to be the most widespread sexually transmitted pathogen among male partners of infertile couples in Sfax (South of Tunisia), as shown by its high prevalence. Our findings confirm previous reports among male partners of infertile couples in Tunisia [41], with lower frequency (15.2% vs 43.3%). This difference might be explained by the use of different methods for the detection of this bacterium. We have used a quantitative real time PCR, which is more specific than in-house PCR-microtiter plate hybridization method. The prevalence of *N. gonorrhoeae* in our study was (5.8%) among male partners of infertile couples. This prevalence was higher than that previously reported in recent studies conducted in other country such as in Canada [42] and in our country [41]. This prevalence of *N. gonorrhoeae* (5.8%) was nearly similar to that reported in other recent studies [43] in Jordan (6.5%). In addition, the qPCR used in our study was reported to be highly sensitive and specific by two authors [44]–[45]. The results of this study, also revealed that the prevalence of *M. genitalium* (3.5%) in infertile men is nearly similar with that reported by Gdoura *et al* (2008) (4.8%) in our country and Al-Sweih *et al* (2012) in Jordan (3.2%) [41]–[46]. Surprisingly, the prevalence of *U. urealyticum* (5.8%) found in our study was considerably lower than previously reported in our country by Gdoura *et al* (2008) [41]. In the literature, the prevalence of *U. urealyticum* in the semen samples of male infertile patients varies from 5% to 42% [47], [48]–[49]. This wide range might be explained by the diversity of detection methods used for characterizing the studied populations. Most of the previous reported studies have discussed the role of *Ureaplasma* in male infertility without discriminating between *U. urealyticum* and *U. parvum* [47]–[50]. In our study, we used a quantitative real time PCR for facilitating the detection and quantification of *U. urealyticum*, *U. parvum*, *M. hominis*, and *M. genitalium* in semen specimens. By this method, *U. parvum* was detected in only one patient (1.1%). The prevalence of this species in our study was lower than that reported by Knox *et al* (2003) (19.2%) and was nearly similar to that reported by Gdoura *et al* (2008) in our country (2.9%) [41]–[48]. In the literature, *M. hominis* has been associated with bacterial vaginosis, pelvic inflammatory disease in women [51]. However, its role in nongonoccocal urethritis and in infertility was rarely investigated [52]. The prevalence of *M. hominis* in our study was (1.1%) comparable to that reported by Rosemond *et al* (2006) (0%) but less than that found by Gdoura *et al* (2008) (9.6%) [41–53]. The role of *C. trachomatis* infection on semen parameters in male infertility is controversial. In fact, a large number of studies have suggested that positive markers for *Chlamydia* infection are not associated with altered sperm parameters [18], [19], [46], [54]–[55]. Others, however, have found that *Chlamydia* infection correlates with reduced sperm motility [34]–[56], increased proportion of sperm abnormalities [57], significant reductions in semen density, sperm morphology, and viability [58] and increased likelihood of leukocytospermia [34]. In addition, Veznik *et al* (2004) reported decreases in seminal plasma, sperm mobility, velocity, and normal morphology in *C. trachomatis*–infected infertile patients compared with those without infection [59]. Mazzoli *et al* (2010) found that *C. trachomatis* affects sperm concentration, percentage of motile sperm and normal morphological forms in patients with prostatitis [12]. A final conclusion from all studies is difficult to establish due to the diversity of population on one hand and variability in sensitivity and specificity of used techniques on the other hand. Moreover, during infertility assessment, infertile couples are not systematically screened for this infection, hence clinically silent *C. trachomatis* infection may be revealed by complications. In fact, the mean duration of infertility in our study was 4 years and patients consulted at different stages of the infection. Lastly, we showed that inoculation of fertile male Swiss mice in the meatus urethra with *C. trachomatis* could lead to alteration of semen parameters (the sperm motility, viability, morphology and sperm concentration) [35]. Our study are concordant with our latter experimental study, the sperm concentration and rapid progressive motility (category a) of spermatozoa in the male partners of infertile couples with *C. trachomatis* DNA in semen specimens showed a significant decrease in comparison with those without infection. Moreover, the sperm vitality and total motility of spermatozoa in the male partners of infertile couples with *C. trachomatis* DNA in semen specimens was lower but without significances compared to patients without infection. The leukocytes count in the male partners of infertile couples with *C. trachomatis* DNA in semen specimens was higher but without significances compared with those without infection. Thus, *C. trachomatis* infection could lead to a decrease in sperm quality.

Apoptosis is a mode of programmed cellular death based on a genetic mechanism that induces a series of cellular, morphological and biochemical alterations, leading the cell to suicide without eliciting an inflammatory response. Mature sperm cells have been reported to express distinct markers of apoptosis-related cell damage [60]–[61]. Externalization of PS to the sperm outer membrane brochure is considered to mark terminal apoptosis. Activated caspase-3, loss of the integrity of the ΔΨm and DNA fragmentation are other markers of terminal apoptosis expressed by a varying proportion of ejaculated sperm [25]–[62]. It has been hypothesized that sperm cell death is associated with male infertility [63]–[64]; however, the exact mechanisms of its involvement remain to be elucidated [65]. Sperm apoptosis and dysfunction have also been reported after sperm exposure to *C. trachomatis* both *in vivo* and *in vitro*. *In vitro* studies have shown that the coincubation of human sperm with *C. trachomatis* serovar E causes a significant decline in the percentage of motile sperm and results in premature sperm death [33]. This sperm death has been demonstrated to be primarily caused by LPS [32]. Moreover, it has been shown that *Chlamydial* LPS interact with CD14 on the sperm surface, leading to increased production of reactive oxygen species and resulting in caspase-mediated apoptosis by using a fluorogenic substrate [29]. Lastly, Satta *et al* (2006) observed that the experimental *C. trachomatis* infection causes sperm PS externalization and DNA fragmentation [30]. *In vivo* studies have reported a higher frequency of sperm cells with fragmented DNA in infertile subjects with *C. trachomatis* genitourinary infection than in control fertile subjects, using the sperm chromatin dispersion test [34]. Moreover, our experimental mouse model has also showed a significant increase of apoptotic and necrotic spermatozoa percentages in infected mice when compared with the control group [35]. In line with these findings, our data demonstrated a direct role of *C. trachomatis* in apoptosis. In order to elucidate the implication of apoptosis in infected semen with *C. trachomatis* DNA, we studied in the first part of our study the viability of spermatozoa using 7-AAD vital stain dye. We found a significant decrease of the mean percentage of viable spermatozoa (7-AAD negative) in male partners of infertile couples with *C. trachomatis* DNA in comparison with uninfected male partners of infertile couples. *C. trachomatis* infection was more correlated negatively with the viability measured using 7-AAD dye than with the viability measured using eosin staining. 7-AAD Dye is more objective than eosin staining. In the second part of our study we studied the state of mitochondrial membrane potential in semen using the lipophilic fluorescent probe JC-1. JC-1 probe has been validated in the assessment of stallion and bull spermatozoa using Flow Cytometry [66]–[67] and provides a more rigorous estimate of metabolic function than Mito Tracker or Rhodamine 123 [67]. In our study, we found a significant increase of the mean percentage of spermatozoa with low ΔΨm in male partners of infertile couples with *C. trachomatis* DNA in semen specimens in comparison with male partners of infertile couples without *C. trachomatis* DNA in semen specimens. At our knowledge, our study represents the first study to characterize the state of ΔΨm in spermatozoa of infertile couples with *C. trachomatis* DNA. In line with our findings, Mabel *et al* (2010) have reported a significant reduction in the percentage of sperm with intact ΔΨm by *in vitro* incubation of human sperm cells with *E. coli* bacteria and the supernatant obtained from these bacteria [68]. In addition, this study demonstrates that contact with *E. coli* bacteria affects sperm mitochondrial function and also confirm the first *in vitro* study reported by Villegas *et al* (2005), demonstrating that soluble factors released by *E. coli* contribute to increase in apoptotic markers in human spermatozoa [69]. Our *in vivo* study confirms these *in vitro* findings and leads to suggest that *C. trachomatis* infection could affect sperm mitochondrial function. Caspase activity has been shown to be present in human sperm [25]–[70]. Furthermore, in infertile men a higher percentage of sperm with activated caspases was found, confirming the existence of a caspase-dependent apoptotic pathway in ejaculated human sperm [71]. In the third part of our study, we studied the activation of caspase 3 in spermatozoa of infertile men. We noticed also a significant increase of caspase 3 activation in male partners of infertile couples with *C. trachomatis* DNA in semen specimens in comparison to male partners of infertile couples without *C. trachomatis* DNA in semen specimens. Our *in vivo* result corroborated with that of Eley *et al* (2005), who demonstrated that the *in vitro* co-incubation of sperm with *C. trachomatis* LPS results in cellular death which is in part due to apoptosis and is caspase 3 mediated [29]. In the last part of our study we studied the sperm DNA fragmentation using (TUNEL) assay. Induction of DNA fragmentation of sperm's nuclei has been widely suggested by several authors because their possible impact on fertility goes beyond fertilization and pregnancy outcome [34]–[72]. In fact, Gallegos *et al* (2008) assessed sperm DNA integrity with sperm dispersion test have found that men with *C. trachomatis* and *Mycoplasma* infections had significantly greater sperm DNA fragmentation than fertile control subjects [34]. These results suggest that *C. trachomatis* and *Mycoplasma* may affect sperm DNA. In line with this study, we noticed a slight increase in sperm DNA damage in male partners of infertile couples with *C. trachomatis* DNA in semen specimens in comparison with male partners of infertile couples without *C. trachomatis* DNA in semen specimens.The limitations of our study were firstly the low number of our population (only 85 infertile men) and secondly the absence of a control groups composed of fertile men. Thus, we have limited our comparison between semen from infected and uninfected infertile men with *C. trachomatis*.

In conclusion, using a quantitative Real time PCR our study indicated that this PCR provides a sensitive measure to detect human *C. trachomatis*, genital *Mycoplasmas*, and genital *Ureaplasmas* DNA, which is useful for epidemiologic studies of these pathogens. Our results also demonstrated that *C. trachomatis* seems to be widespread among male partners of infertile couples in Sfax (South of Tunisia). This study supports that *C. trachomatis* infection could lead to a decrease in sperm quality and apoptosis induction. In fact, *C. trachomatis* infection was found to increase the ΔΨm dysfunction in spermatozoa and caspase 3 activation. However, sperm DNA damage was not significantly associated to *C. trachomatis* infection. This leads us to suggest that caspase 3 could be implicated during *C. trachomatis* infection but does not cause directly DNA damage.
